# The impact of physician–nurse task shifting in primary care on the course of disease: a systematic review

**DOI:** 10.1186/s12960-015-0049-8

**Published:** 2015-07-07

**Authors:** Nahara A. Martínez-González, Ryan Tandjung, Sima Djalali, Thomas Rosemann

**Affiliations:** Institute of Primary Care, University of Zurich, University Hospital of Zurich, Pestalozzistrasse 24, CH-8091 Zurich, Switzerland

**Keywords:** Systematic review, Physician–nurse substitution, Task shifting, Course of disease, Randomized controlled trials, Health policy

## Abstract

**Background:**

Physician–nurse task shifting in primary care appeals greatly to health policymakers. It promises to address workforce shortages and demands of high-quality, affordable care in the healthcare systems of many countries. This systematic review was conducted to assess the evidence about physician–nurse task shifting in primary care in relation to the course of disease and nurses’ roles.

**Methods:**

We searched MEDLINE, Embase, The Cochrane Library and CINAHL, up to August 2012, and the reference list of included studies and relevant reviews. All searches were updated in February 2014. We selected and critically appraised published randomized controlled trials (RCTs).

**Results:**

Twelve RCTs comprising 22 617 randomized patients conducted mainly in Europe met the inclusion criteria. Nurse-led care was delivered mainly by nurse practitioners following structured protocols and validated instruments in most studies. Twenty-five unique disease-specific measures of the course of disease were reported in the 12 RCTs. While most (84 %) study estimates showed no significant differences between nurse-led care and physician-led care, nurses achieved better outcomes in the secondary prevention of heart disease and a greater positive effect in managing dyspepsia and at lowering cardiovascular risk in diabetic patients. The studies were generally small, of varying follow-up episodes and were at risk of biases. Descriptive details about roles, qualifications or interventions were also incomplete or not reported.

**Conclusion:**

Trained nurses may have the ability to achieve outcome results that are at least similar to physicians’ for managing the course of disease, when following structured protocols and validated instruments. The evidence, however, is limited by a small number of studies reporting a broad range of disease-specific outcomes; low reporting standards of interventions, roles and clinicians’ characteristics, skills and qualifications; and the quality of studies. More rigorous studies using validated tools could clarify these findings.

**Electronic supplementary material:**

The online version of this article (doi:10.1186/s12960-015-0049-8) contains supplementary material, which is available to authorized users.

## Resumen

**Antecedentes:**

La transferencia de tareas del médico a profesionales de enfermería en atención primaria atrae enormemente a los legisladores de salud. Esta estrategia promete abordar la escasez de recursos humanos y demandas de atención asequible y de alta calidad en los sistemas de salud de muchos países. Realizamos una revisión sistemática para evaluar la evidencia sobre la transferencia de tareas médico-enfermería en atención primaria con relación al curso de la enfermedad y a las funciones de enfermería.

**Métodos:**

Realizamos búsquedas en los registros de Medline, Embase, Cochrane Library y CINAHL, hasta Agosto del 2012; y en las listas de referencia de los estudios incluidos y de revisiones sistemáticas relevantes. Actualizamos las búsquedas en Febrero del 2014. Seleccionamos y realizamos una valoración crítica de los ensayos controlados aleatorios (ECA) ya publicados.

**Resultados:**

Doce ECA realizados principalmente en Europa con 22 617 participantes asignados al azar reunieron los criterios de inclusión. Profesionales de enfermería, principalmente enfermeras médico, dieron el cuidado de atención, mayormente mediante el uso de protocolos estructurados e instrumentos validados. Se reportaron veinticinco medidas únicas del curso de la enfermedad. La mayoría (84 %) de éstas mostraron diferencias no significativas entre grupos, pero el profesional de enfermería consiguió tasas de pacientes significativamente mejores que el médico en la prevención secundaria de enfermedades cardiacas, un efecto positivo mayor en el manejo de dispepsia, y en la reducción del riesgo cardiovascular en pacientes diabéticos. Los estudios fueron generalmente pequeños, de seguimiento variado, y vulnerables a sesgos de selección, detección y deserción. Descripciones de las funciones, calificaciones e intervenciones no se reportan o son incompletas.

**Conclusión:**

Profesionales de enfermería capacitados podrían obtener resultados de similitud al médico en el manejo del curso de la enfermedad, mediante el seguimiento de protocolos estructurados e instrumentos validados. La evidencia, sin embargo, se limita a un pequeño número de estudios que reportan una amplia gama de resultados de enfermedades específicas, y un bajo estándar en el reporte de intervenciones, características y funciones del profesional clínico, habilidades y calificaciones; y por la calidad de los estudios. Estudios con mejor calidad de métodos, que utilicen herramientas validadas podrían aclarar estos hallazgos.

## Background

Chronic illness and disability are major contributing factors of morbidity and mortality worldwide. Long life spans and ageing populations are already imposing a major challenge for the infrastructures of healthcare systems [1]. These factors and patients’ expectations give rise to professional care-giving demands, consequently increasing the need for more activity in the community, ambulatory and primary care. There is currently a global shortage of 7.2 million physicians, nurses and midwives, however, and this number is estimated to increase to about 12.9 million by 2035 [2].

A popular approach to overcome this increasing shortage of human resources is task shifting, a process of delegation whereby tasks are moved to less specialized healthcare workers [3]. The strategy aims to efficiently and effectively reorganize the existing healthcare human resources to improve the distribution of workload, increase service capacity and reduce healthcare costs [4, 5]. Physician–nurse task shifting is carried out by transferring specific functions or tasks traditionally from the domain of physicians to nurses. Nurses are less costly to employ and train than physicians and are one of the largest groups of qualified healthcare providers [6]. In many countries, nurses are being granted more responsibilities and a wider range of clinical tasks [7]. This means that nurses might have a key role in medical areas where workforce shortages are a major health issue. Chronic conditions, for example, depend on resourceful caregiving, a long-term plan, continuous monitoring and adherence to disease management to achieve better health outcomes [8].

Two reviews suggested that nurses can provide same quality care and achieve as-good health outcomes as primary care physicians, but the volume and methodological quality of studies were insufficient [9, 10]. Measures of the course of disease such as symptoms, severity and complications were rarely reported. These measures help in monitoring the course of disease and treatment response and can have a major impact on patient outcomes; some may serve as surrogate measures of disease severity. We systematically reviewed the evidence on the effectiveness of physician–nurse task shifting in primary care in relation to measures of the course of disease and nurses’ roles.

## Methods

The protocol of our review followed a population, intervention, comparison and outcome (PICO) approach [11] and the recommended guidelines [12] for the reporting of systematic reviews and meta-analyses (see Additional file 1).

### Study identification

We searched MEDLINE (OVID), Embase (Elsevier), CINAHL (EBSCOHost) and The Cochrane Library of Systematic Reviews (Wiley) which includes the Cochrane Effective Practice and Organization of Care Group. The searches were not restricted by age, publication date or country and included a study design filter for randomized controlled trials (RCTs) and a combination of MeSH terms, text words, free text terms and synonyms for “primary care”, “skill mix”, “doctors”-“nurse” “substitution” (see Additional file 2). Additional publications were identified by manual searches of the reference lists of included studies and relevant reviews. All searches were carried out to cover publications from the inception of databases to August 2012 and were updated in February 2014.

### Study inclusion and exclusion criteria

We included peer-reviewed RCTs published in English that examined task shifting from physicians to nurses in primary care (general practices, community or ambulatory care settings). Studies were eligible if care from family physicians, paediatricians and/or geriatricians was compared to care delivered by nurses (nurse-led care) in all roles under a task-shifting model of care and for patients of all ages and all conditions, and if studies reported outcome measures related to the course of disease including symptoms, severity and complications. We excluded measures of quality of life, satisfaction, mortality, hospital admissions, process of care or clinical parameters that were not surrogates of the course of disease.

We focused on a task-shifting approach by differentiating it from supplementation based on the framework from a Cochrane review [10]. Under task shifting, clearly delineated tasks or functions traditionally from the domain of physicians are transferred to nurses [3]. Nurses may receive specific or competency-based training to perform such tasks/functions and would deliver consultations with autonomous or delegated responsibility. Studies of task shifting thus compare the performance between nurses (as main figure of care) and physicians when both manage the same work or tasks in a similar capacity. In a supplementation approach, nurses complement the work of physicians or extend the range of services to improve the quality of care. Studies of supplementation compare nurses working alongside other clinicians (multi-professional service) with physicians working alone (uni-professional service). We excluded studies of supplementation.

### Study selection and quality assessment

Two authors independently screened titles and abstracts and assessed the full text of eligible publications and the methodological features of included studies, resolving differences by consensus. Based on well-established guidelines [13], we assessed the methodological features of studies including core items of quality criteria that could influence the risk of bias (sequence and allocation concealment, blinding of participants, personnel and outcome assessors and intention to treat (ITT)). Following the debate about the validity of scores for the assessment of risk of bias [14, 15], we did not calculate a composite score. We describe the studies’ adequacy in each item with an overall judgement of the quality of evidence.

### Data abstraction

Structured data collection forms were used independently by two authors to abstract the bibliographic details, population demographics, interventions (training competency, roles, type of care, clinical responsibility, use of guidelines, follow-up length) and outcome data (in all forms, for example, binary, continuous and/or semi-quantitative) including the length of consultations in minutes. Differences were resolved through consensus. Based on the studies’ description of interventions and competencies, we categorized nurses into the following: nurse practitioner (NP) or NP with additional degree/courses (NP+), for example, NP with Master degree; registered nurse (RN); or licensed nurse (LN). Outcomes were grouped according to disease area, for example, heart disease for heart failure and coronary heart disease. Data from a single study reported across various publications were extracted as one unit. Publications reporting more than one RCT were extracted as separate studies.

### Data synthesis

Data were analysed based on the individual trial estimates. Using Review Manager (Version 5.1) [16], we calculated the unadjusted relative risks (RRs) or the weighted mean differences (WMDs) and 95 % confidence intervals (CI) of the absolute end points. There was mostly one study per outcome which precluded the ability to perform meta-analyses. We tabulated the effect sizes, and the results were synthesized narratively. We considered *P* < 0.05 to imply statistical significance. Following established techniques [17], standard deviations were estimated using the information from the studies’ statistical analyses.

## Results

### Study identification

**Fig. 1:**
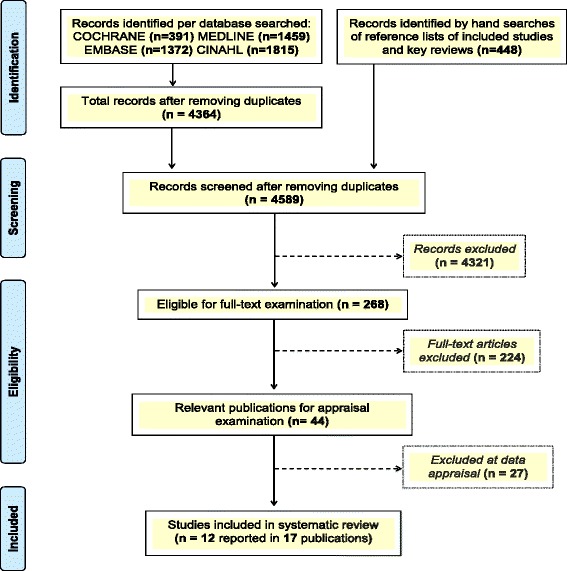
Illustration of the study selection process

Figure 1 shows the flow of study selection. Our literature searches identified 4 589 original records. Based on screening of titles and abstracts, 268 records were eligible for detailed examination of full-text publications; 44 of these were relevant for appraisal, but we excluded 27 for the reasons provided in Additional file 3. Twelve RCTs reported in 17 publications [18–34] met the inclusion criteria.

### Study and population characteristics

Table 1 provides the summary characteristics of studies in review including the study design, setting, population demographics and interventions (see Additional file 4 for a detailed description). The trials were conducted in the UK (*n* = 6), the Netherlands (*n* = 3), South Africa (*n* = 2) and Russia (*n* = 1). More than one follow-up episode was reported in a few cases; median of maximum follow-up was 6 (range: 0.5–48) months. The 12 trials comprised of 22 617 enrolled or randomized patients (range: 100–9 252); 4 had less than 200 patients (range: 100–175). Mean age in 10 trials and ranged from 35 (SD 9.63) to 69.5 (SD 10.6) years; median age was 27.6 (IQR: 9.0–44.9) in another trial, and patients’ age ranged from 0 to >75 years in another one, and 34.7 % of the population were male.

**Table 1:** Summary characteristics of studies included in review

Study			Setting	Population	Nurse group				Physician group				Intervention							
First author, publication (*y*)	Location	Design, period^a^	Facilities, *n*	Diagnosis	Nurses (*n*)	Patients (*N*)	Mean age (SD), *y*	Male, %	Phys., (*n*)	Patients (*N*)	Mean age (SD), *y*	Male, %	By	FCA	GDL	1st C	UV	OC	C, *n*	FUP, months
Fairall et al., 2012 [25]	ZA 2	cRCT2, 2008–2010	Nurse ART clinic, 31	HIV/AIDS	103	3029	38.0 (8.9)	30	nr	3202	38.0 (9.6)	27	LN	n	y	n	n	y	>1	12–18
Fairall et al., 2012 [25]	ZA 1	cRCT1, 2008–2010	Nurse ART clinic, 31	HIV/AIDS	103	5390	36.0 (9.6)	33	nr	3862	35.0 (9.6)	31	LN	n	y	n	n	y	>1	12–18
Houweling et al., 2011 [27]	NL 3	RCT, period nr	Practice, 1	DM2	2	116	67.1 (11.0)	53	5	114	69.5 (10.6)	42	NP	y	y	n	n	y	>1	14
Andryukhin et al., 2011 [18]	RU 1	RCT, 2006–2009	Medical centre practice, 1	Heart failure with preserved ejection fraction	10	50	66.5 (3.2)	27	8	50	68.0 (4.3)	34	NP/LN	n	y	n	n	y	>1	6
Dierick-van Daele et al., 2009 [24]	NL 2	RCT, 2006	Practice, 15, Reference, 5	Common complaints	12	817	42.8 (16.5)	38	50	684	46.1 (16.6)	40	NP+	n	y	y	n	n	1	0.5
Chan et al., 2009 [22]	UK 6	RCT, 2002–2004	Nurse clinic, 1	GORD, moderate gastritis, dyspepsia after direct access gastroscopy	nr	89	50.2 (13.9)	49	nr	86	48.4 (12.8)	49	NP+	n	y	n	n	y	>1	6
Hesselink et al., 2004 [26]	NL 1	RCT, 1998–2002	Practice, 12	Asthma and COPD	2	139	49.9 (14.2)	35	14	137	44.7 (13.6)	28	LN	n	y	n	n	y	>1	0.5, 12, 24
Denver et al., 2003 [23]	UK 5	RCT, 2000–2001	Nurse hospital-based hypertension clinic, *n* = nr	DM2,hypertension, under blood pressure lowering treatment	nr	60	58.1 (13.8)	57	nr	60	62.4 (9.1)	70	NP+	n	y	n	n	y	>1	6
Kernick et al., 2000 [28]	UK 4	RCT, period nr	Health centre, 1	Psoriasis and eczema	1	55	47.4 (18.4)	39	nr	54	51.7 (15.8)	48	NP+	n	y	n	n	y	>1	4
Kinnersley et al., 2000 [34]	UK 3	RCT, period nr	Practice, 10	Diverse conditions	12	1465^b^	range: 0–>75	39	10	1465^b^	range: 0–>75	42	NP	n	nr	y	y	n	1	0.5
Shum et al., 2000 [32]	UK 2	RCT, 1998–1999	Practice, 5	Acute minor illnesses	5	900	IQR:26.0 (9.0–41.7)	40	19	915	IQR:29.1 (9.7–44.9)	40	NP	n	nr	y	y	n	≥1	0.5
Campbell et al., 1998 [19–21, 29–31, 33]	UK 1	RCT, 1995–1996	Practice, 19	CHD secondary prevention	28	673	65.9 (7.9)	58	nr	670	66.3 (8.3)	58	NP	n	y	n	n	y	>1	12, 48

Studies are listed by year (*y*) of publication, in decreasing order

Phys. (*n*): number of physicians; Nurses (*n*): number of nurses; Patients (*N*): number of patients enrolled (Fairall et al. 2012 [25]) or randomized; SD: standard deviation; UK: United Kingdom; NL: The Netherlands; ZA: South Africa; RU: Russia; RCT: randomized controlled trial; cRCT: cluster randomized controlled trial; Facilities *n*: number of facilities; DM (2): diabetes mellitus (type 2); GORD: gastro-esophageal reflux disease; COPD: chronic obstructive pulmonary disease; CHD: coronary heart disease; IQR: interquartile ranges; NP: nurse practitioner; NP+: nurse practitioner with additional degrees/courses; LN: licensed nurse; FCA: full clinical autonomy; GDL: guideline/(semi-structured)protocol-based interventions; 1stC.: 1st contact; UV: urgent visits; OC: ongoing care; C (*n*): number of consultations; FUP: length of follow-up; y: yes; n: no; nr: not reported

^a^Start and end year when studies were conducted

^b^Number of randomized patients per group not reported

Task shifting was carried out in general practices, nurse clinics and healthcare centres, for a wide range of possible diagnoses (diverse, minor acute, common or specific, for example, hypertension) in patients requiring single-contact care, single-contact and urgent care and/or ongoing care. The tasks varied widely from assessment, history taking, preparation, diagnostic, monitoring and prescription to decisions on eligibility for and initiation of treatment, referral, follow-up and secondary prevention. Description of nurses’ competencies and training components often lacked enough detail. The number of participating nurses and physicians was reported in 10 and 6 trials; the median number was 11 (range: 1–103) and 12 (range: 5–50), respectively. Nurses performed roles of NPs in eight trials, LNs in three trials and LNs and NPs in one trial. Nurses’ and physicians’ experience was reported in four (range: 1–12 years) and one (16 years) trials, respectively. Three trials stated the nurses’ educational degree: a Masters in Advanced Nursing, a diploma in general practice and a special degree in patient education. Nurses delivered independent interventions in all trials but required support from physicians in 11 trials and assumed full responsibility in 1 trial. All but two trials reported the use of clinical guidelines or protocols.

### Risk of bias in individual studies

The methodological assessment per quality item for each trial is reported on Table 2. Ten trials individually assigned patients to intervention groups and two randomized 31 clinics with 103 nurses. No trial fulfilled all the quality criteria assessed. Ten trials reported patient inclusion and exclusion criteria and seven measured the intervention by definition of a primary outcome. The trials were at risk of selection bias since only six had adequate random sequence generation and allocation concealment. No trial reported blinding of patients and providers, and three performed blinded assessment of outcomes, making trials vulnerable to performance and detection biases. Eleven trials reported to have calculated sample sizes, eight had at least 20 % attrition (overall range: %: 24–59) and seven reported the use of intention-to-treat techniques to deal with missing data. Nine trials reported the funding sources; one was industry funded.

**Table 2:** Methodological features of included studies

Study		Inclusion and exclusion criteria	Outcome		Sequence generation	Allocation concealment	Blinding	Sample		Attrition, %	Funding
First author	Location		1ry	2ry				calc.	size		
Fairall et al., 2012 [25]	ZA 2	✓	✓	✓	A	A	^d^	✓^e^	≥200	<20	G
Fairall et al., 2012 [25]	ZA 1	✓	✓	✓	A	A	^d^	✓^e^	≥200	≥20	G
Houweling et al., 2011 [27]	NL 3	✓	✓	✓	I	A	NP	✓	≥200	<20	G
Andryukhin et al., 2011 [18]	RU 1	✓			U	I	^c^	✓^e^	<200	≥20	None
Dierick-van Daele et al., 2009 [24]	NL 2	✓			A	A	NP		≥200	≥20	G
Chan et al., 2009 [22]	UK 6	✓			A	A	^b^	✓	<200	<20	nr
Hesselink et al., 2004 [26]	NL 1	^a^	✓	✓	U	U	^b^	✓	≥200	≥20	nr
Denver et al., 2003 [23]	UK 5	^a^	✓	✓	I	I	NP	✓^e^	<200	<20	nr
Kernick et al., 2000 [28]	UK 4	✓	✓		A	U	U	✓^e^	<200	≥20	Ind.
Kinnersley et al., 2000 [34]	UK 3	✓	✓	✓	A	A	NP	✓^e^	≥200	≥20	G
Shum et al., 2000 [32]	UK 2	✓			A	A	NP	✓^e^	≥200	≥20	G
Campbell et al., 1998 [19–21, 29–31, 33]	UK 1	✓			A	I	^b^	✓	≥200	≥20	G

Studies are listed by year (*y*) of publication, in decreasing order. A tick indicates the specific criteria fulfilled

Blinding: whether patients, care providers and/or outcome assessors were blinded; UK: United Kingdom; NL: the Netherlands; ZA: South Africa; RU: Russia. I: inadequate; A: adequate; U: unclear; NP: not performed; G: government; Ind.: industry; P: private; nr: not reported

^a^Inclusion criteria only

^b^Blinding of outcome assessors

^c^Single blinding

^d^Data analysts partly blinded

^e^Intention to treat strategies to deal with missing data

### Outcomes

Twenty-five different measures of the course of disease were reported for a wide range of conditions (Table 3). All but three [24, 26] of the measures were taken using validated methods, and the follow-up time span varied widely.

**Table 3:** Effect estimates of studies in review

Study		Nurses’ role	Population diagnosis	Outcome	Measurement method/scale	FUP, months	Nurse group		Physician group		Effect estimate
First author, *y*	Location										
Binary data							*n*	*N*	*n*	*N*	RR (95 % CI)
Heart disease											
Andryukhin et al., 2011 [18]	RU 1	NP/LN	Heart failure PEF	Positive^b^ changes in class HF	NYHA	6	18	40	8	35	1.97 (0.98 to 3.96)
Campbell et al., 1998 [19–21, 29–31, 33]	UK 1	NP	CHD secondary prevention	Chest pain	ATyPeS/SF36^a^	12	232	508	250	498	0.91 (0.80 to 1.04)
Campbell et al., 1998 [19–21, 29–31, 33]	UK 1	NP	CHD secondary prevention	Chest pain	ATyPeS/SF36	48	147	430	129	385	1.02 (0.84 to 1.24)
Campbell et al., 1998 [19–21, 29–31, 33]	UK 1	NP	CHD secondary prevention	Worsening chest pain	ATyPeS/SF36	12	37	519	54	500	0.66 (0.44 to 0.98)
Campbell et al., 1998 [19–21, 29–31, 33]	UK 1	NP	CHD secondary prevention	Worsening chest pain	ATyPeS/SF36	48	44	439	35	395	1.13 (0.74 to 1.73)
Lung disease											
Hesselink et al., 2004 [26]	NL 1	LN	Asthma/COPD	No chronic cough and phlegm production or an improvement	Present/absent	12	43	108	39	85	0.87 (0.63 to 1.20)
Hesselink et al., 2004 [26]	NL 1	LN	Asthma/COPD	No chronic cough and phlegm production or an improvement	Present/absent	24	41	93	33	79	1.06 (0.75 to 1.49)
Hesselink et al., 2004 [26]	NL 1	LN	Asthma/COPD	No wheezing or an improvement in frequency	Never, ever, most days and night	12	68	106	51	85	1.07 (0.85 to 1.34)
Hesselink et al., 2004 [26]	NL 1	LN	Asthma/COPD	No wheezing or an improvement in frequency	Never, ever, most days and night	24	53	93	37	79	1.22 (0.91 to 1.63)
Infectious disease											
Fairall et al., 2012 [25]	ZA 2	LN	HIV/AIDS	Suppressed viral load in patients receiving ART^d^	Viral load	12–18	2156	3029	2230	3202	1.02 (0.99 to 1.06)
Fairall et al., 2012 [25]	ZA 1	LN	HIV/AIDS	Suppressed viral load in patients starting ART^d^	Viral load	12–18	1706	2375	1062	1449	0.98 (0.94 to 1.02)
Diverse, acute and minor conditions											
Shum et al., 2000 [32]	UK 2	NP	Acute and minor	Same, improved or cured self-reported health status	Murphy	0.5	650	672	646	661	0.99 (0.97 to 1.01)
Kinnersley et al., 2000 [34]	UK 3	NP	Diverse conditions	Same or improved symptoms (much better, better or unchanged)	Likert-type and single reminders	0.5	472	484	515	529	1.00 (0.98 to 1.02)
Continuous data							Mean (SD)	*N*	Mean (SD)	*N*	WMD (95 % CI)
Lung disease											
Hesselink et al., 2004 [26]	NL 1	LN	Asthma/COPD	Mean change score in dyspnea	MRCQT^c^	12	0.00 (1.3)	115	0.10 (1.3)	94	−0.10 (−0.45 to 0.25)
Hesselink et al., 2004 [26]	NL 1	LN	Asthma/COPD	Mean change score in dyspnea	MRCQT^c^	24	0.20 (1.4)	96	0.30 (1.3)	80	−0.10 (−0.50 to 0.30)
Metabolic disease											
Denver et al., 2003 [23]	UK 5	NP+	DM2 with hypertension, under BPLT	Mean fall in 10-year CHD risk	Framingham CHD risk score	6	−2.33 (3.87)	59	−0.33 (2.16)	56	−2.00 (−3.14 to −0.86)
Denver et al., 2003 [23]	UK 5	NP+	DM2 with hypertension, under BPLT	Mean fall in 10-year stroke risk	Framingham stroke risk score	6	−4.33 (6.0)	59	−1.80 (3.53)	56	−2.53 (−4.32 to −0.74)
Digestive disease											
Chan et al., 2009 [22]	UK 6	NP+	GORD, moderate gastritis	Mean score, dyspepsia severity	GDSS (Gladys) score^g^	6	4.90 (2.9)	89	7.20 (3.1)	86	−2.30 (−3.19 to −1.41)
Skin disease											
Kernick et al., 2000 [28]	UK 4	NP+	Psoriasis/eczema	Mean score for symptoms and severity of skin condition	Self-evaluation clinical score^e^	4	7.60 (3.3)	35	8.1 (3.3)	46	−0.50 (−1.95 to 0.95)
Common complaints											
Dierick-van Daele et al., 2009 [24]	NL 2	NP+	Common complaints	Mean difference in the degree of burden of illness	LikertQT^f^	0.5	−1.77 (3.18)	473	−1.50 (2.63)	451	−0.27 (−0.65 to 0.11)
Dierick-van Daele et al., 2009 [24]	NL 2	NP+	Common complaints	Mean difference in the concerns about illness	LikertQT^f^	0.5	−1.51 (3.20)	476	−1.40 (2.97)	450	−0.11 (−0.51 to 0.29)
Qualitative data											
Metabolic disease											
Houweling et al., 2011 [27]	NL 3	NP	DM2	Perceived burden of DM symptoms and rating of symptom troublesomeness	Type 2 Diabetes Symptom Checklist^h^	14	“significant differences at follow-up for some of the Diabetes Symptom Score dimensions (data not shown). After 14 months, the mean sub-dimension scores for DM symptoms ‘fatigue’ and ‘cognitive distress’ and the total scores were lower in each group, although no difference was observed between the groups.”				
Lung disease											
Hesselink et al., 2004 [26]	NL 1	LN	Asthma/COPD	Respiratory complaints within two weeks after intervention	Disturbance (present/absent) for >1 day or night	0.5	“no significant group differences in the number of days or nights disturbed, OR 0.96 (95 % CI 0.56 to 1.61)”				
Common complaints											
Dierick-van Daele et al., 2009 [24]	NL 2	NP+	Common complaints	Complications due to illness	Mean number of days of work absence	0.5	“1.11 days (nurse, SD 0.32; physician, SD 0.31) of work absence in average”				
Dierick-van Daele et al., 2009 [24]	NL 2	NP+	Common complaints	Complications due illness	Mean number of days of inability for daily activities	0.5	“no statistically significant differences between groups; mean days unable for daily activities: nurse-led care 2.53 (SD 2.89), physician-led care 2.69 (SD 2.90)”				

Studies are listed in order of increasing length of follow-up, within each category of outcomes

UK: United Kingdom; NL: the Netherlands; ZA: South Africa; RU: Russia; *n*: number of patients or number of events; *N*: total number of patients per group; SD: standard deviation; RR: relative risk; WMD: weighted mean difference; CI: confidence intervals; DM(2): diabetes mellitus (type 2); GORD: gastro-esophageal reflux disease; COPD: chronic obstructive pulmonary disease; CHD: coronary heart disease; BPLT: blood pressure lowering treatment; PEF: preserved ejection fraction; MRCQT: Medical Research Council Questionnaire; LikertQT: Likert-type questionnaire; GDSS: Glasgow Dyspepsia Severity Score (Gladys); NYHA: New York Heart Association Functional Classification; ATyPeS: Angina TyPe specification scale

^a^ATyPeS is designed to use with the SF36 questionnaire to assess the presence, frequency and course of chest pain

^b^Positive changes mean regression of class or stay within class I of NYHA

^c^MRCQT ranking from 0 (no dyspnea) to 4 (very serious); positive mean values in each group indicate improvement; mean differences with negative values mean a reduction or improvement

^d^ZA1 trial: patients starting ART whose results were available for at least 6 months. ZA2 trial: 76 % and 78 % of the patients in the intervention and control group, respectively, had been receiving ART for at least 2 years at the time of viral load measurements

^e^Three out of eight possible symptoms, each ranked from 1 (mild) to 5 (very severe). The sum score gave a clinical score from 3 (best state) to 15 (worst)

^f^Likert-type questionnaire ranking from 0 (excellent) to 10 (poor/worse); mean differences with negative values mean a reduction or improvement

^g^GDSS (Gladys) ranking from 0 (no symptoms) to 20 (symptoms)

^h^34-item scale based on yes/no questionnaires for perceived burden of symptoms including hyperglycemic, hypoglycemic, cardiac, neuropathic, psychological and vision-related. Summary responses ranked from 1 (symptom not occurred or not perceived as troublesome) to 5 (symptom extremely troublesome) on Likert-type scale

### Heart disease

In one trial, NPs delivered care techniques to facilitate behavioural change following clinical guidelines to perform secondary prevention of coronary heart disease (CHD) [20]. In a time span of 12 or 48 months, nurse-led care showed no significant differences to physician-led care in the number of patients with chest pain, reflected on the Angina TyPe specification scale and the SF-36 questionnaire. In a time span of 12 months, however, nurse-led care showed significantly fewer patients (7.1 %), compared to physician-led care (10.8 %), who reported worsening (little or much worse) chest pain (RR 0.66, 95 % CI 0.44 to 0.98). This difference levelled off at 48 months.

In another trial, LNs and/or NPs delivered patient education, treatment, exercise and training information and counselling following clinical guidelines to manage heart failure with preserved ejection fraction [18]. In a time span of 6 months, nurse-led care showed no significant differences to physician-led care in the number of patients with positive changes in heart failure class, reflected on the New York Heart Association Functional Classification tool: positive changes meant regression of class or stay within class I.

### Lung disease

In one trial, LNs delivered patient education and followed structured protocols to manage asthma and chronic obstructive pulmonary disease [26]. In a time span of 12 and 24 months, both nurse-led care and physician-led care showed a small (mean change) improvement in dyspnea scores, but the differences were not significant, reflected on the Medical Research Council Questionnaire. The trial also showed no significant differences between groups in the number of patients in whom chronic cough, phlegm production and wheezing were absent or improved and in the number of days or nights disturbed.

### Metabolic disease

In one trial, NPs+ followed clinical guidelines to manage hypertension in patients with type 2 diabetes mellitus (DM2); no other details about the interventions delivered were reported [23]. In a time span of 6 months, nurse-led care compared to physician-led care showed that the differences in mean fall from baseline were significantly lower for stroke risk (WMD −2.53, 95 % CI −4.32 to −0.74) and CHD risk (WMD −2.00, 95 % CI −3.14 to −0.86), reflected on the Framingham 10-year stroke risk and the 10-year CHD risk scores.

In another trial, NPs followed clinical guidelines to manage patients with DM2; no other details about the interventions delivered were reported [27]. The trial stated that in a time span of 14 months significant differences with nurse-led care were observed for some of the DM2-related symptom score dimensions, reflected on the revised version of DM2 Symptom Checklist. The mean sub-dimension scores for fatigue, cognitive distress and the total scores were stated to be lower in each group.

### Digestive disease

In one trial, NPs+ ran a follow-up clinic and managed gastroscopy and dyspepsia according to guidelines; no other details about the interventions delivered were reported [22]. In a time span of 6 months, nurse-led care significantly improved (maintained or reduced) dyspepsia, compared to physician-led care, reflected in the Glasgow Dyspepsia Severity Score (Gladys) (WMD −2.30, 95 % CI −3.19 to −1.41).

### Skin disease

In one trial, NPs+ managed and prescribed medications for the management of psoriasis and eczema following a dermatology manual; no other details about the interventions delivered were reported [28]. In a time span of 4 months, nurse-led care showed no significant differences to physician-led care in the severity of skin condition and symptoms for psoriasis and eczema, reflected in a summary clinical score for self-evaluation of symptoms.

### Infectious disease

In one trial, LN assessed eligibility and prepared patients for antiretroviral therapy (ART); monitored, prescribed and initiated ART therapy; and referred patients to physicians for ART initiation and re-prescriptions and followed clinical guidelines for the management of HIV [25]. In a time span of 12–18 months, nurse-led care compared to physician-led care showed no significant differences in the number of patients for whom suppressed viral load was maintained while they were under ART; 77 % of the patients had ART for at least 2 years. In another trial, nurses delivered the same interventions for HIV patients whose viral load results were available for at least 6 months [25]. In a time span of 12–18 months, there were no significant differences between groups in the number of patients for whom suppressed viral load was maintained after starting ART.

### Diverse, acute, minor or common complaints

In one trial, NPs performed history taking and physical examinations, gave advice and prescribed treatment and referrals, for the management of acute and minor illness [32]. In another trial, NPs performed same-day consultations for diverse conditions; no other details about the interventions delivered were reported [34]. Both trials included children (Table 1). None of the trials reported whether nurses followed clinical guidelines to deliver interventions. In a time span of half a month, the two trials showed no significant differences between groups in the number of patients who reported their health status as same, improved or cured, reflected on the Murphy scale [32] and a Likert-type scale [34].

In another trial, NPs+ assessed symptoms, performed clinical examinations and diagnosis, planned further treatment, prescribed and performed referrals to primary or secondary care, ordered clinical tests and examinations and followed clinical guidelines for the management of common complaints [24]. In a time span of half a month, nurse-led care compared to physician-led care showed no significant (mean) differences (before and 2 weeks after consultation) in the degree of burden of illness and in the concerns about illness, reflected on a Likert-type questionnaire. The trial also stated no significant differences between groups in the mean number of days for which patients reported being unable to perform daily activities due to illness. The stated average number of days that patients missed their job due to illness was 1.11 days.

### Length of consultations

Four trials [24, 27, 32, 34] reported the length of consultations; all showed longer consultations by nurses than by physicians (WMD range: 1.90–3.80 min; 95 % CI: 1.32 to 4.26).

The quality of studies was mixed, and sample sizes were generally small. The three trials [20, 22, 23] in which the intervention effects favoured nurse-led care were of lower methodological quality (small study (*N* < 200), lack/unclear random generation and allocation concealment and/or blinding and/or ≥20 % attrition). Among these, only one [22] was superior in quality but used a small patient population. A trial [25] of somehow higher quality (*N* > 3000, better random generation and allocation concealment, <20 % attrition, some blinding) also showed no significant differences between groups. Of the remaining trials, four [24, 25, 32, 34] had larger patient populations and better random generation and allocation concealment, but did not fulfil blinding and/or attrition criteria.

## Discussion

In this systematic review, we identified 12 trials aiming to compare physician-led care with nurse-led care under a task-shifting model in primary care. Nurse-led care was provided mainly by nurse practitioners whose scope expanded to a wide range of clinical domains and patient populations. Nurses performed various tasks with different degrees of clinical responsibility. They used clinical guidelines and validated tools in most studies to deliver care and monitor and identify disease-specific changes of the course of disease. The volume of literature continues to be low, however, and belongs mostly to European high-income countries, mainly the UK and the Netherlands. The evidence represents a wide range of disease-specific measurements of disease progression, is at risk of biases and consists of variable follow-up episodes. Nevertheless, we found that nurse-led care was statistically not significantly different to physician-led care in 84 % of the patient outcomes reported. The remaining 16 % significantly favoured nurse-led care compared to physician-led care. Nurses may thus have the ability to meet the needs of healthcare systems with an increasing shortage of physicians, in keeping with reports calling for nurses to be used more and to perform greater roles [7, 9, 10, 35].

The use of structured protocols that combine non-pharmacological interventions with pharmacological therapy and the use of validated tools in 75–84 % of the studies might have resulted in better or similar care by nurses than by physicians. It has been shown [36, 37] that through delivering interventions that include education, counselling and advice, nurses may encourage more life changes and retain more patients in treatment resulting in greater medication compliance, improve prevention and better symptom control. Interventions that include the provision of information about causes of illness and patients’ disease, for example, in the trial of patients with dyspepsia, may have also added benefits to nurse-led care resulting in more motivated patients and more effective (self-care) interventions in general. Research has shown that patients generally want more information than they routinely receive from healthcare professionals and that they like greater involvement in the process of making decisions about their treatment [38]. Good communication skills can also increase adherence to treatment and outcome effectiveness [39, 40] and may have led to more patients being involved in their own care and treatment decisions.

The use of disease-specific protocols may have also guided nurse-led care interventions to at least similar effectiveness as physicians-led care in the management of stroke and CHD risk in diabetic patients. Disease-specific protocols that include intensified non-pharmacological programmes have indeed been associated with improved cardiovascular risk factors but also with patient self-efficacy and patient safety [41–43]. Using these protocols may have also led nurses to successfully perform therapy techniques that require safe (for example, inhalation) and continuous management for conditions such as COPD and asthma control. In addition, appropriate identification and monitoring of disease changes have been recommended to successfully engage and retain patients in care [44, 45]. Following like-guidelines, nurses might have appropriately quantified and monitored viral load, for example, resulting in similar effectiveness to physician-led care at maintaining suppressed viral load. In all, nurses might have followed protocols more strictly resulting in outcome improvements [46].

The long-term nature of chronic disease necessitates a programme with an appropriate time span. It is unclear, however, if the length of follow-up was adequate to identify a true effect; the studies do not report details about the monitoring length needed to reflect a change. For most of the studies, the time span was short, 6 months or less. Disease complexity may have also resulted in longer consultations by nurses than by physicians. In all studies, nurses were trained and/or took courses for delivering the studies’ interventions. Further, nurses were supported by physicians in most of the studies, suggesting of the potential benefits of collaborative teams in the improvement of patient outcomes, for example, reducing modifiable risk factors [47, 48]. In a broader context, by participating in these studies, nurse-led care patients may have become more aware of their condition and were thus more receptive to care resulting in better outcomes.

It is uncertain to what extent nurses’ educational preparation and type of nurses’ roles influenced the outcome effects. The three trials that reported the nurses’ educational degree showed no significant differences. Although nurses were in NP roles in 16 % (4/25) of the outcomes that favoured nurse-led care, the studies were of lower methodological quality somehow (lack/unclear random generation and/or allocation concealment and/or ≥20 % attrition and/or small study). Furthermore, nurses were NPs and/or LNs in 84 % (21/25) of the outcomes which showed no significant differences. Although these studies were of variable quality, one trial of somehow higher quality also had a bigger sample (*N* > 3000).

Future research could benefit from a more rigorous methodology and better study reporting. Change in health status over a time interval can occur due to the natural course of disease and/or as a result of the care provided. Future studies should therefore report about the regularity of disease monitoring needed to obtain an improved outcome, consist of longer follow-up episodes and continue using validated instruments. This could lead to increased safety, effectiveness and improved compliance to medication [36, 37]. Self-report scales should be considered more carefully since these may not be able to accurately detect the causes for the changes in the course of disease. In order to understand how nurses’ competencies and qualifications influence patient outcomes and quality of care, future studies should map all available nurses’ roles and consistently report the educational qualifications and clinical responsibilities, training and clinicians’ characteristics (for example, nurse–physician–patient ratios and years of experience). Reporting of intervention components as recently recommended [49] could help to identify the benefits of this strategy. The implementation of non-pharmacological strategies should be further considered to improve disease management and quality outcomes [50, 51].

### Strengths and limitations of this systematic review

This is, to our knowledge, the first task-shifting systematic review with a focus on the study of measurements of the course of disease. Although many measures were reported, most were unique to each study precluding the incorporation of meta-analyses. Where data were available, however, we report the trial estimates. Although observational studies if well implemented could provide important information, we only included RCTs because these are at a lower risk of bias and allow the estimation of causal effects. A small number of studies met the inclusion criteria, possibly because the number of studies in this area is increasing slowly. We did not search for grey literature, and we excluded publications reported in languages other than English. We used thorough electronic and manual searches, however, and screened relevant reviews (some in foreign languages) to identify all relevant publications. It was difficult to understand the level of nurses’ autonomy since nurses were supported by physicians in most studies. In most cases, the interventions are not described in detail, and we could not make clear judgments about nurses’ educational degree.

## Conclusion

Trained nurses, mostly NPs, appeared to achieve outcomes of at least similar effects as physicians for the management of disease progression in a wide range of patient populations. Structured protocols and validated tools might be some of the main boosters of outcome improvement. The implementation of non-pharmacological and patient-centred care approaches may also lead to successful nurse-led care interventions. A clear definition of roles, qualifications, skills and experience, essential for an effective and safe transfer of tasks and functions, is only reported in low standards. It is therefore unclear to what extent nurses should be involved in task shifting from physicians. The evidence is also limited by the mixed methodological quality of the trials, although a few of the trials have larger patient populations. More good quality studies using validated tools and larger samples from many countries should improve the reporting standards and consistency of nurses’ roles, qualifications and interventions.

## Additional files

Additional file 1:
**PRISMA checklist.**
Additional file 2:
**Search strategy in Ovid MEDLINE.** Note: Similar search strategies were performed and run in Embase, The Cochrane Library of Systematic Reviews and CINAHL and include specific search filters for RCTs. Additional file 3:
**Studies excluded based on appraisal of full-text articles.**
Additional file 4:
**Population and interventions in the included studies.** Note: Studies are listed by year (y) of publication, in decreasing order. UK, United Kingdom; NL, The Netherlands; ZA, South Africa; RU, Russia; RCT, randomized controlled trial; cRCT, cluster randomized controlled trial; m, months; n/r, not reported; ART, antiretroviral therapy; HbA1c, haemoglobin; CD4, T-cell surface glycoprotein CD4.
