# Supplementary material for: The impact of physician–nurse task shifting in primary care on the course of disease: a systematic review
Source: Hum Resour Health. 2015 Jul 7;13:55. doi: 10.1186/s12960-015-0049-8 (PMC4493821; doi:10.1186/s12960-015-0049-8)
Supplement: Additional file 2: — Search strategy in Ovid MEDLINE. Note: Similar search strategies were performed and run in Embase, The Cochrane Library of Systematic Reviews and CINAHL and include specific search filters for RCTs. [file 12960_2015_49_MOESM2_ESM.doc]

**Additional file 2:** Search strategy in Ovid Medline*.

| **#** | **Searches** | **Results** |
| --- | --- | --- |
| 1 | exp General Practice/ or exp Primary Health Care/ or exp Private Practice/ or Family Physicians/ or general practitioners/ or physicians, primary care/ or exp geriatrics/ or Geriatric Assessment/ or exp pediatrics/ | 223536 |
| 2 | exp Nursing Care/ or Primary Nursing/ or Community Health Nursing/ or Family Nursing/ or Nursing, Practical/ or Advanced Practice Nursing/ or exp Geriatric Nursing/ or exp Pediatric Nursing/ | 129054 |
| 3 | exp Ambulatory Care/ or ambulatory care facilities/ or community health centers/ or maternal-child health centers/ or outpatient clinics, hospital/ or pain clinics/ or surgicenters/ | 75627 |
| 4 | (primary adj2 (health?care$ or care$ or medic$)).ti,ab,hw. | 96885 |
| 5 | ((family adj2 (physician$ or doctor or practitioner or practice or internist or medic$)) or (general adj2 (internist or physician$ or doctor or practitioner or practice or medic$ or care$ or health$care$)) or (personal adj2 (doctor or physician$)) or (physician$ adj2 (practitioner or practice)) or (internal adj medicine) or geriatri$ or paediatri$ or pediatri$).ti,ab,hw,mp. | 459697 |
| 6 | ((community or ambulatory or shared) adj4 (care$ or health$care) adj4 (facility or facilities or service$ or cent$ or clinic$)).ti,ab,hw. | 15372 |
| 7 | or/1-6 | 713736 |
| 8 | exp Physician Assistants/ or nurse clinicians/ or nurse practitioners/ | 23718 |
| 9 | (nurs$ adj2 (family or primary or care$ or practitioner or practice or clinic$ or regist$ or specialist$ or leader or consultant$ or physician$ or expert or district or advanced or assessment or visit$ or geriatri$ or paediatri$ or pediatri$)).tw,mp. | 150356 |
| 10 | ((nurs$ adj2 appropriately adj trained) or (nurs$ adj2 community adj2 health adj2 care) or (nurs$ adj2 first contact) or (assistan$ adj2 (physician$ or medic$ or health$care$)) or (clinic$ adj2 support) or (clinic$ adj2 (nurse$led or nurse led))).ti,ab,hw. | 20215 |
| 11 | or/8-10 | 175262 |
| 12 | Nurse's Practice Patterns/ | 553 |
| 13 | delegation, professional/ | 360 |
| 14 | professional autonomy/ | 8143 |
| 15 | Clinical Competence/ | 58713 |
| 16 | exp Professional Role/ | 61186 |
| 17 | 12 or 13 or 14 or 15 or 16 | 120249 |
| 18 | (((substitut$ or transfer$ or swap or replac$) adj3 (((doctor$ or GP or GPs or physician$ or practi$ or general) adj2 practitioner$) or job or role or task$ or skill$ or perform$ or responsibility or autonom$)) or ((delegat$ or supervis$) adj5 (responsibility or performance$ or role$ or job or tasks)) or (autonom$ adj (professional or responsibility or self$regulation)) or (clinical adj skill$ adj competence) or (((skill$mix or skill$) adj mix$) or skill$) or (role$ adj4 (advance or chang$ or enhanc$ or expan$ or transfer$)) or (team$ adj4 (patient care or multidisciplinary or cooperation) adj4 autonom$)).ti,ab,hw. | 150274 |
| 19 | 17 or 18 | 254445 |
| 20 | 7 and 11 and 19 | 14767 |
| 21 | (letter or letter$).pt,sh. or (editorial or historical article or anecdote or commentary or note or case report$ or case study).pt. or (editorial or historical article or anecdote or commentary or note or case report$ or case study).pt. or (animal studies or animals, laboratory or experimental animal or animal experiment or animal model or rodentia or rodents or rodent).sh. | 2816007 |
| 22 | (randomi?ed controlled trial or controlled clinical trial).pt. or (randomi?ed or placebo or randomly or trial or groups).ab. | 1748897 |
| 23 | exp cluster analysis/ or cross-over studies/ or ((cluster$ adj2 random$) or (communit$ adj2 intervention$) or (communit$ adj2 random$)).mp. | 74125 |
| 24 | ((non$equivalent adj3 control$) or posttest$ or post test$ or post-test$ or pre test$ or pretest$ or pre-test$ or quasi-experiment$ or quasi experiment$ or quasiexperiment$ or timeseries or time series or time-series or (time adj2series adj2 analysis) or (interrupted adj2 time adj2series)).mp. | 34416 |
| 25 | 22 or 23 or 24 | 1807744 |
| 26 | 25 not 21 | 1780623 |
| 27 | 20 and 26 | 1376 |
| 28 | limit 27 to humans | 1348 |

**Note:** ^*^Similar search strategies were performed and run in EMBASE, The Cochrane Library of Systematic Reviews and CINAHL and include specific search filters for RCTs.
