# Supplementary material for: The impact of physician–nurse task shifting in primary care on the course of disease: a systematic review
Source: Hum Resour Health. 2015 Jul 7;13:55. doi: 10.1186/s12960-015-0049-8 (PMC4493821; doi:10.1186/s12960-015-0049-8)
Supplement: Additional file 3: — Studies excluded based on appraisal of full-text articles. [file 12960_2015_49_MOESM3_ESM.doc]

**Additional file 3:** Studies excluded based on appraisal of full-text articles.

| **No.** | **References of studies** | **Reason for exclusion** |
| --- | --- | --- |
|  | Blanchard MR, Waterreus A, Mann AH (1995) The effect of primary care nurse intervention upon older people screened as depressed. Int J Geriatr Psychiatry 10: 289-298. | Cohort study and multidisciplinary team approach. |
|  | Blanchard MR, Waterreus A, Mann AH (1999) Can a brief intervention have a longer-term benefit? The case of the research nurse and depressed older people in the community. Int J Geriatr Psychiatry 14: 733-738. | Nurse working in close collaboration with other clinicians. |
|  | Cave AJ, Wright A, Dorrett J, McErlain M (2001) Evaluation of a nurse-run asthma clinic in general practice. Primary Care Respiratory Journal 10: 65-68. | Not an intervention comparison between nurses and physicians. |
|  | Dierick-Van Daele ATM, Steuten LMG, Metsemakers JFM, Derckx EWCC, Spreeuwenberg C, et al. (2010) Economic evaluation of nurse practitioners versus GPs in treating common conditions. British Journal of General Practice 60: 28-33. | Outcomes of interest not reported. |
|  | Du Moulin MFMT, Hamers JPH, Paulus A, Berendsen CL, Halfens R (2007) Effects of introducing a specialized nurse in the care of community-dwelling women suffering from urinary incontinence: a randomized | Outcomes of interest not reported. |
|  | Flynn BC (1974) The effectiveness of nurse clinicians' service delivery. Am J Public Health 64: 604-11. | Outcomes of interest not reported. |
|  | Hemani A, Rastegar DA, Hill C, al-Ibrahim MS (1999) A comparison of resource utilization in nurse practitioners and physicians. Eff Clin Pract 2: 258-65. | Outcomes of interest not reported. |
|  | Hiss RG, Armbruster BA, Gillard ML, McClure LA (2007) Nurse care manager collaboration with community-based physicians providing diabetes care: a randomized controlled trial. Diabetes Educator 33: 493-502. | Outcomes of interest not reported. |
|  | Hollinghurst S, Horrocks S, Anderson E, Salisbury C (2006) Comparing the cost of nurse practitioners and GPs in primary care: modelling economic data from randomised trials. British Journal of General Practice 56: 530-5. | Outcomes of interest not reported. |
|  | Jarman B, Hurwitz B, Cook A, Bajekal M, Lee A (2002) Effects of community based nurses specialising in Parkinson's disease on health outcome and costs: randomised controlled trial. BMJ 324: 1072-5. | Outcomes of interest not reported. |
|  | Kernick D, Powell R, Reinhold D (2002) A pragmatic randomised controlled trial of an asthma nurse in general practice. Primary Care Respiratory Journal 11: 6-8. | Outcomes of interest not reported. |
|  | Krein SL, Klamerus ML, Vijan S, Lee JL, Fitzgerald JT, et al. (2004) Case management for patients with poorly controlled diabetes: a randomized trial. Am J Med 116: 732-739. | Nurse working in close collaboration with other clinicians based on a chronic care model. |
|  | Kuethe M, Vaessen-Verberne A, Mulder P, Bindels P, van Aalderen W (2011) Paediatric asthma outpatient care by asthma nurse, paediatrician or general practitioner: Randomised controlled trial with two-year follow-up. Primary Care Respiratory Journal 20: 84-91. | Outcomes of interest not reported. |
|  | Lapointe F, Lepage S, Larrivee L, Maheux P (2006) Surveillance and treatment of dyslipidemia in the post-infarct patient: can a nurse-led management approach make a difference? Can J Cardiol 22: 761-767. | Intervention (telephone) not of interest for this review and not part of usual care interventions of physicians. |
|  | Leenders F, Beusmans G, Swerts H, editors (2006) A practice nurse for patients with cardiovascular disease, an explorative study. | Report in Dutch. Version of article in English was not found. |
|  | Lenz ER, Mundinger MO, Kane RL, Hopkins SC, Lin SX (2004) Primary care outcomes in patients treated by nurse practitioners or physicians: two-year follow-up. Med Care Res Rev 61: 332-51. | Outcomes of interest not reported. |
|  | Lewis CE, Resnik BA, Schmidt G, Waxman D (1969) Activities, events and outcomes in ambulatory patient care. N Engl J Med 280: 645-649. | Observational study and outcomes not of interest for this review. |
|  | Lewis CE, Resnik BA (1967) Nurse clinics and progressive ambulatory patient care. N Engl J Med 277: 1236-41. | Outcomes of interest not reported. |
|  | Mundinger MO, Kane RL, Lenz ER, Totten AM, Tsai WY, et al. (2000) Primary care outcomes in patients treated by nurse practitioners or physicians: a randomized trial. JAMA 283: 59-68. | Outcomes of interest not reported. |
|  | Sackett DL, Spitzer WO, Gent M, Roberts RS (1974) The Burlington randomized trial of the nurse practitioner: health outcomes of patients. Ann Intern Med 80: 137-142. | No real substitution. At least 30% of patients in both groups were seen by the physicians at the end of study and data was not split into mutually exclusive groups. |
|  | Spitzer WO, Sackett DL, Sibley JC, Roberts RS, Gent M, et al. (1974) The Burlington randomized trial of the nurse practitioner. N Engl J Med 290: 251-256. | No real substitution. At least 30% of patients in both groups were seen by the physicians at the end of the study and data was not split into mutually exclusive groups. |
|  | Tonstad S, Alm CS, Sandvik E (2007) Effect of nurse counselling on metabolic risk factors in patients with mild hypertension: a randomised controlled trial. European Journal of Cardiovascular Nursing 6: 160-164. | No real substitution. In both experimental and control groups the nurse provides interventions and different stages of care. |
|  | Van Son L, Vrijhoef, H. (2004) Supporting the general practitioner. A randomized controlled trial investigation the effects of a practice nurse on asthma, COPD, and diabetes. Huisarts en wetenschap: 15-21. | Report in Dutch. Version of article in English was not found. |
|  | Venning P, Durie A, Roland M, Roberts C, Leese B (2000) Randomised controlled trial comparing cost effectiveness of general practitioners and nurse practitioners in primary care. BMJ 320: 1048-53. | Outcomes of interest not reported. |
|  | Voogdt-Pruis HR, Beusmans GHMI, Gorgels APM, Kester ADM, Van Ree JW (2010) Effectiveness of nurse-delivered cardiovascular risk management in primary care: A randomised trial. British Journal of General Practice 60: 40-46. | Outcomes of interest not reported. |
|  | Williams KS, Assassa RP, Cooper NJ, Turner DA, Shaw C, et al. (2005) Clinical and cost-effectiveness of a new nurse-led continence service: a randomised controlled trial. Br J Gen Pract 55: 696-703. | Nurse working in close collaboration with other clinicians. Control group received care from nurses, physicians and specialists and data was not split into mutually exclusive groups. |
|  | Winter C (1981) Quality health care: patient assessment [MSc]. Long Beach, CA: California State University. | Outcomes of interest not reported. |
