# Supplementary material for: The impact of physician–nurse task shifting in primary care on the course of disease: a systematic review
Source: Hum Resour Health. 2015 Jul 7;13:55. doi: 10.1186/s12960-015-0049-8 (PMC4493821; doi:10.1186/s12960-015-0049-8)
Supplement: Additional file 4: — Population and interventions in the included studies. Note: Studies are listed by year (y) of publication, in decreasing order. UK, United Kingdom; NL, The Netherlands; ZA, South Africa; RU, Russia; RCT, randomized controlled trial; cRCT, cluster randomized controlled trial; m, months; n/r, not reported; ART, antiretroviral therapy; HbA1c, haemoglobin; CD4, T-cell surface glycoprotein CD4. [file 12960_2015_49_MOESM4_ESM.doc]

**Additional file 4:** Participants and interventions in the included studies.

| **Study** | | **Patients** | | **Nurse intervention** | | |
| --- | --- | --- | --- | --- | --- | --- |
| **First author, year** | **Geographical location** | **Included** | **Excluded** | **Training/competency, guidelines, tasks** | **Nurses’ responsibilities and nurses / physicians experience (years)** | **Follow-up, months** |
| Fairall et al, 2012 [24]. | ZA 2 | Adults with HIV who received ART for at least six months with ongoing treatment at the time of enrollment; clinics with more than 100 eligible patients used a random sample taken electronically (proportional to the total number of eligible patients); in other clinics all eligible patients were included. | Patients who did not return to their clinic after enrollment or who were potentially exposed to the intervention; deaths before trial started or were relocated to another clinic. | Nurse-led care based on African guidelines. Middle nurse managers trained to assume responsibility for ART: assess and prepare patients, initiate, monitor and prescribe ART or referral to physicians for ART initiation and re-prescriptions. Physicians referred patients diagnosed with HIV to nurse-led clinic to establish eligibility for ART. | For phase 2 and phase 3: same as in ZA1.  Experience n/r. | 12-18 |
| Fairall et al, 2012 [24]. | ZA 1 | Adults with HIV participating in an ART program who had CD4 counts of ≤350/µl and had not yet started ART: either eligible for ART (if CD4 counts were ≤200 cells per μL) or likely to become eligible during the trial (if CD4 counts were 201-350 cells per μL). | Patients who did not return to their clinic after enrollment because they needed to visit a clinic more than once to initiate ART after obtaining CD4 results; who started ART before trial started, who deaths before trial started or were relocated to another clinic. | Nurse-led care based on African guidelines. Middle nurse managers trained to assume responsibility for ART: assess and prepare patients; initiation, monitoring and prescribing ART or referral to physicians for ART initiation and re-prescriptions. Physicians referred patients diagnosed with HIV to nurse-led clinic to establish eligibility for ART. | For phase 2: nurses assumed responsibility for repeating ART prescriptions in stable (median, m: 3.5, range: 1-35) patients.  For phase 3: nurses assumed responsibility for initiating ART in selected patients (median, m: 30.5, range: 0-32) and nurses referred patients who did not meet above criteria.  Experience n/r. | 12-18 |
| Houweling et al, 2011 [26]. | NL 3 | Patients with Diabetes Mellitus type 2 treatment and under medication, with hba1c (Hemoglobin levels) measurements within the last 3 years. | Patients with Diabetes Mellitus type 2 not being treated in primary care setting, inability to participate because of age, comorbidities or in the opinion of general practitioner, not willing to return to follow-up. | Practice nurse with one week training in Diabetes Mellitus to manage transferred patients based on guidelines. | Full responsibility.  Experience n/r. | 14 |
| Andryukhin et al, 2011 [17]. | RU 1 | Patients of at least 50 years of age with heart failure with preserved ejection fraction, informed consent. | Patients with blood pressure of <90/60 mmHg or >160/100 mmHg, under optimal antihypertensive therapy, acute coronary syndrome within previous six months, significant valvular stenosis, insulin diabetes mellitus dependent, confirmed chronic obstructive pulmonary disease, conditions limiting participation in the rehabilitation (see reference for more details). | Nurse-led care based on Russian National guidelines. Nurses with special degree in patient education obtained in joint course: patient education, treatment and exercise training information and counselling. | Prescription of medication and non-pharmacological measures (diet, alcohol intake, weight reduction, smoking cessation, activity and exercise training) provided by physician.  Experience n/r. | 6 |
| Dierick-van Daele et al, 2009 [23]. | NL 2 | Patients with common complaints aged ≥16 who sought a general practitioner for initial consultation. | Patients with unregistered in practice, language or reading problems, or with reason for appointment not provided. | Practice nurse with Master degree in Advance Nursing, trained in common complaints to manage patients based on guidelines (assess symptoms, perform physical examinations and diagnosis; decisions on further treatment, prescribing, referrals to 1ry and 2ry services, ordering clinical tests and investigations). | Prescriptions and referrals had to be validated by physician.  Experience  Nurses: 12 years (SD 7.6) as senior nurses in general practice; 0.17 years as nurse practitioners. Physicians: 16 years. | 0.5 |
| Chan et al, 2009 [21]. | UK 6 | Patients with mild gastro-esophageal reflux disease or moderate gastritis referred to gastroscopy for evaluation. | Patients with sinister symptoms (dysphagia, vomiting, anemia, rapid weight loss, history of gastric surgery, severe gastroscopy findings e.g. Peptic ulcer, tumor, esophagitis grade C/D, Barrett’s esophagus, anatomical abnormality). | Gastrointestinal practice nurse to manage Dyspepsia based on Guidelines and to run follow-up clinic for consultations following gastroscopy. | Authorized to adopt treatment according to Guidelines and performed specific tests (e.g. Breath urea, barium meal).  Experience n/r. | 6 |
| Hesselink et al, 2004 [25]. | NL 1 | Patients with Asthma, Chronic Obstructive Pulmonary Disease or mixed disease, 16-75 years old, with symptoms (cough, phlegm or dyspnea) within year before study, with current use of Chronic Obstructive Pulmonary Disease or asthma medication. | Patients with presence of other pulmonic disease, terminal disease. | Physicians’ assistant to manage patients based on semi-structured protocols: patient education on asthma and Chronic Obstructive Pulmonary Disease. | No full responsibility (details n/r).  Experience  Nurses: 2 years as GP assistants.  Physicians: n/r. | 0.5, 12, 24 |
| Denver et al, 2003 [22]. | UK 5 | Patients with Diabetes Mellitus type 2, previous diagnosis of hypertension, or who were in receipt of Blood Pressure Lowering Treatment. | Patients with life to threatening comorbidities requiring intensive management. | Hypertension nurse to manage hypertension based on clinical guidelines. | No full responsibility (details n/r).  Experience n/r. | 6 |
| Kernick et al, 2000 [27]. | UK 4 | Patients with diagnosis of psoriasis or eczema, 18-65 years old and a minimum of three repeated prescriptions for a topic medication in the past year. | n/r. | Practice nurse with training in management of psoriasis and eczema and to deliver care based on dermatology manual: management and prescription of medications. | Prescriptions required a doctor's signature if recommendations were in line with guidelines.  Experience n/r. | 4 |
| Kinnersley et al, 2000 [33]. | UK 3 | Patients with diverse complaints requesting same day appointments, informed consent. | Patients with patients seemingly too ill to wait or unable to understand the research, women seeking emergency contraception. | Nurse practitioner with a nurse diploma on care for same day consultations for primary care. | Physicians were always available to prescribe when necessary.  Experience  Nurses: at least 1 year working as nurse practitioner and regularly seeing patients requesting same day consultations.  Physicians: n/r. | 0.5 |
| Shum et al, 2000 [31]. | UK 2 | Patients with minor illnesses aged ≥1 years, who requested and were given appointment on the same day, informed consent. | Patients with pregnancy problems, severe chest or abdominal pain, severe breathing problems, vomiting blood, fits or blackouts, psychiatric problems, literacy or language difficulties. | Nurse practitioner who had no specific experience in seeing patients with minor illnesses but took a course on managing minor illnesses and were piloted before the study: management, history taking, physical examinations, advice and treatment, prescribing and referral. | Prescriptions required a doctor's signature.  Experience  Nurses: 8.4 years (SD3.8) in practice nursing.  Physicians: n/r. | 0.5 |
| Campbell et al, 1998 [18-20, 28-30, 32]. | UK 1 | Patients with coronary heart disease. | Patients with terminal illness, dementia, housebound patient, explicit request to see general practitioner. | Health visitors, district and practice nurses with training in clinic protocols/guidelines and techniques to facilitate behavioral change: secondary prevention of coronary heart disease. | No full responsibility to manage patients.  Experience n/r. | 12, 48 |

**Note:** Studies are listed by year (y) of publication, in decreasing order.

UK, United Kingdom; NL, The Netherlands; ZA, South Africa; RU, Russia; RCT, Randomized Controlled Trial; cRCT, cluster Randomized Controlled Trial; m, months; n/r = not reported; ART, Antiretroviral Therapy; HbA1c, Hemoglobin; CD4, t-cell surface glycoprotein CD4.
